# Microglial Response to Inflammatory Stimuli Under Electromagnetic Field Exposure

**DOI:** 10.26502/acbr.50170467

**Published:** 2025-06-30

**Authors:** Yssel Mendoza-Mari, Marija Stojanovic, Dan E. Miulli, Devendra K. Agrawal

**Affiliations:** 1 Department of Translational Research, College of Osteopathic Medicine of the Pacific, Western University of Health Sciences, Pomona, CA, USA; 2 Department of Neurosurgery, Riverside University Health System Medical Center, Moreno Valley, Pomona, CA, USA

**Keywords:** Brain injury, Electromagnetic field, HMC3 cells, Microglia, TNF-α, Traumatic Brain Injury

## Abstract

Microglial cells constitute the largest number of non-neuronal cells in the brain. As part of their immune surveillance function, they are responsible for detecting the presence of both external and internal danger signals, stimulating a defense response through the release of pro-inflammatory cytokines. Once the damage is controlled, microglia stimulate a reparative response that allows tissue homeostasis to be maintained. When this balance is not physiologically achieved, the use of drugs or other non-pharmacological therapies is needed to promote tissue repair and prevent the appearance of complications secondary to the primary damage. In the particular case of traumatic brain injury (TBI), the application of low frequency electromagnetic field (EMF) has proven very helpful in reducing the levels of inflammatory mediators. In the present study we investigated the effect of EMF in an “*in vitro*” model of tumor necrosis factor alpha (TNF-α)-induced neuroinflammation. Human microglial cells (HMC3) were treated with TNF-α (50 ng/mL) and, after 20 minutes, were exposed to 2.5 or 5 Hz EMF for 3 min. The effect of both treatments on survival, migration capacity and transcriptional expression of M1/M2 phenotypic markers was evaluated at 6, 24 and 48 hours. The exposure to EMF increased the survival rate of cells incubated with high doses of TNF-α and significantly reduced the migration rate of TNF-α treated cells. The analysis of expression patterns in different time points showed that EMF promoted the expression of M1 and M2 phenotypic markers in a time-dependent manner, suggesting a stimulating effect on the phagocytic capacity of microglial cells. Further studies are necessary to fully characterize the effects of EMF on the function of primary microglial cells within a brain injury-like microenvironment.

## 1. Introduction

Traumatic brain injury (TBI) is one of the leading causes of mortality and disability worldwide [1]. After mechanical trauma, the brain goes through two phases of damage. The first phase involves focal and diffuse damage, hematomas, and hemorrhages. During the second phase, a series of molecular events occur that can extend over time and are primarily responsible for the development of long-term complications. One of these molecular events is neuroinflammation. This process arises as a natural defensive response to trauma, and it involves the activation of local and circulating immune cells and the expression of pro-inflammatory molecules, all of them in charge of removing damaged tissue and pathogens [2]. Although physiological inflammation is necessary to promote brain recovery and healing, persistent neuroinflammation can result in additional injury [3]. There are different types of cells involved in this process but, undoubtedly, microglial cells play a leading role as they are the primary immune cells in the brain [4].

In physiological conditions, microglial cells are responsible for immune surveillance, phagocytosis of apoptotic debris, maintaining synaptic homeostasis, and production of growth factors that modulate neuronal activity [5]. But in response to tissue trauma, microglia are activated by pathogen associated molecular patterns (PAMPs) or damage-associated molecular patterns (DAMPs) released from damaged tissue, and also by pro-inflammatory cytokines such as interferons (INF-γ), interleukins, or tumor necrosis factor (TNF-α) [6]. Microglial cells undergo morphological and functional changes, among which the production of pro-inflammatory / anti-inflammatory cytokines stands out. This response is essential in regulating the balance between neuroinflammation and tissue repair.

There are some distinctive markers that contribute to identifying microglia from monocyte/macrophage cells. Among them, purinergic receptor P2RY12 and transmembrane protein 119 (Tmem119) are considered the most specific general microglia markers [7]. There are other biomarkers like ionized calcium-binding adapter molecule 1 (IBA-1), cluster of differentiation receptors (CD68, CD11b, CD14, CD45, CD80, and CD115), fractalkine receptor (CX3CR1), ferritin, F4/80, high-affinity immunoglobulin epsilon receptor subunit gamma (FCER1G) and vimentin. These markers are less specific as they are also expressed by other cell types [8].

After activation, microglial phenotypes can be classified in M1 (pro-inflammatory) and M2 type (anti-inflammatory). Particularly, M2 phenotype has 3 subtypes: M2a, M2b, and M2c [9]. Microglial phenotypes can be identified by their morphology and the expression of typical markers and cytokine secretion patterns. M1 cells have an amoeboid shape, round, and large cell bodies, while M2 cells have small cell bodies and distal branches. As antigen presenting cells, activated microglia expresses MHC II molecules, as well as other co-stimulatory receptors like CD86. Other nonspecific transmembrane and surface proteins expressed by activated microglia are CD68, CD14, CA115, CX3CR1, F4/80, and FCER1G [8]. CD16 and CD32, membrane receptors for the Fc region of IgG, are considered specific markers for M1 phenotype [10], as well as increased levels of CD86, CD40 and CD45. On the other hand, classic markers for anti-inflammatory M2 phenotype are CD206, a receptor localized in cellular and endosomal membranes [11] and the hemoglobin scavenger receptor CD163 [12]. More specific sub phenotypes M2a, M2b and M2c simultaneously express markers for both M1 and M2, but there is no consensus about markers for each subtype [13].

It has been shown that after TBI, the ratio of M1 to M2 microglia is highly shifted towards pro-inflammatory polarization [14], so any treatment aimed to reduce or revert this unbalance could highly contribute to minimize neuroinflammation. Among the non-pharmacological approaches to diminish short- and long-term complications of TBI, the application of Electromagnetic field (EMF) has emerged as a very promising treatment [15]. Several molecular mechanisms have been proposed to illustrate the different effects of EMF in different cell types and organ systems [16–18]. Our group recently showed that the application of a low frequency EMF application preserved the neuronal tissue morphology and reduced inflammatory markers at the transcriptional and translational levels in a swine model of TBI [19]. Besides, EMF also showed to stimulate genes related to immune cell infiltration, myelination, reactive oxygen species regulation, thyroid hormone transportation, cell proliferation, and cell migration, all of them contributing to increase the repairing process after trauma [20,21]. Based on these results, in this study we aim to elucidate the specific effect of EMF on microglial cells by analyzing two of their main characteristics: the expression of specific markers of M1 and M2 phenotypes and their migration capacity in the presence of pro-inflammatory damage. These *in vitro* results might contribute to explaining the effects observed *in vivo*.

To accomplish our goal, we performed the study in the HMC3 cells [22,23]. This microglial cell line has been extensively used in different studies and characterized after the stimulation with stressors like lipopolysaccharide, INF-γ or different interleukins [23]. In our study, we recreated the pro-inflammatory milieu of TBI by incubating the cells in presence of human recombinant TNF-α, a key mediator of the molecular pathways activated after trauma [24]. To analyze the effect of EMF, we applied the treatments 20 minutes after the addition of stressor, based on our previous results that immediate application of EMF after TBI is associated to a better resolution of inflammation [19]. Our results showed that EMF increased the survival rate of cells incubated with high doses of TNF-α and significantly reduced the migration rate of TNF-α treated cells. The analysis of expression patterns in the different time point showed that EMF promoted the expression of M1 and M2 phenotypic markers in a time dependent way, suggesting a stimulating effect on the phagocytic capacity of microglial cells.

## 2. Materials and Methods

### 2.1 Cell culture:

Human microglial cells (HMC3; CRL-3304, ATCC, Manassas, VA, USA) were cultured in Eagle’s Minimum Essential Medium (EMEM) (ATCC^®^ 30–2003) supplemented with 10% fetal bovine serum (FBS) (Phoenix Research, Swedesboro, NJ, USA) and 1X antibiotic-antimycotic (penicillin, streptomycin, amphotericin B) (Gibco, Carlsbad, CA, USA) at 37°C and 5% CO_2_. During maintenance of the cells, the culture medium was replaced every 2 days. All experiments were carried out at cell passages <10.

### 2.2 Electromagnetic field application:

For the application of EMF, the plates were placed under a helmet provided with induction sensors (model BS-1000, Quasar Federal Systems, San Diego, CA) and dual-layered Mu-metal (MuMETAL^®^, Magnetic Shield Corporation, Bensenville, IL) previously described [25–27]. Stimulation thresholds of 2.5 or 5 Hz with 1 V signal intensity were evaluated. In all experiments 3 min exposure to EMFs were applied 20 min after the addition of TNF-α to the cultures. After this time, plates were returned to their normal incubation conditions at 37°C and 5% CO_2_.

### 2.3 Viability assay:

Cell viability was evaluated by 3-(4,5-dimethylthiazol-2-yl)-2,5-diphenyltetrazolium bromide (MTT) assay. The HMC3 cells were seeded in 96-well plates at a density of 1×10^4^ cells/well, 24 h prior to the addition of the treatments to ensure their adherence to the plastic. Cells were treated with recombinant human TNF-α (25–200 ng/mL) (300-01A-50UG, ThermoFisher Scientific, Waltham, MA, USA). After 20 h, 20 μL of 5 mg/mL MTT (M6494, ThermoFisher Scientific, Waltham, MA, USA) sterilely prepared in phosphate buffered saline (PBS) were added into each well. Cells were incubated for another 4 h, culture medium was removed from the wells and 200 μL/well of dimethyl sulfoxide (DMSO) (D2650, Sigma Aldrich, St. Louis, MO, USA) was added to dissolve the insoluble formazan crystals. Cell viability was measured using a Multimode Microplate Reader BioTek Synergy Neo2 (Agilent, Santa Clara, CA, USA) at 570 and 620 nm wavelengths. Results were expressed as the percentage of cell viability compared to untreated cells. A similar analysis was conducted to verify whether EMF modifies the survival rates of HMC3 cells.

### 2.4 Scratch migration assay:

HMC3 cells were seeded in six-well plates at a density of 6×10^5^ cells/well, 24 h prior to the addition of treatments. After this time, 200 μL sterile tips were used to gently scratch the cell monolayers in the largest diameter of the well in vertical and horizontal positions. Cell debris was removed by washing the cells with sterile PBS 1X. Fresh EMEM culture medium supplemented with TNF-α (50 ng/mL) was added to the cells during 20 min. Untreated cells were used as control. After this time, the cells were subjected to EMF as previously described, no EMF was considered as control. Wound areas were measured at 6, 12, 24, and 45 hours after the addition of the treatments using Fiji Image J Software (version 1.54J, NIH, USA) [28]. The wound closure was estimated as the percentage of initial area using the following formula: % = (A_*tn*_ / A_*t0*_) × 100% where A_*tn*_ represents the area in a given time point and A_*t0*_ is the initial area.

### 2.5 Quantitative Real-Time Polymerase Chain Reaction (RT-qPCR):

HMC3 cells were seeded and incubated with TNF-α as described in the scratch assay. qPCR analysis was performed at 6, 24 and 48 h after the addition of treatments. Cell lysis was performed using TRIZOL (T9424, Millipore Sigma, Burlington, MA, USA) following the manufacturer’s instruction protocol in our laboratory. RNA pellets were resuspended in 30 μL of nuclease-free water (BP561-1, ThermoFisher Scientific, Waltham, MA, USA) and RNA yield was quantified using Nanodrop 2000 Spectrophotometer (Thermo Fisher, Waltham, MA, USA). Two micrograms of total RNA were used to synthesize complementary DNA (cDNA) using AzuraQuant^™^ cDNA Synthesis Kit (AZ-1996, Azura Genomics Inc., Raynham, MA, USA) according to manufacturer’s instruction using a T100^™^ Thermal Cycler (Bio-Rad Laboratories, Hercules, CA, USA). The cDNAs were diluted 1:20 in nuclease-free water and qPCR reactions were prepared in a final volume of 10 μL and in triplicate using AzuraView^™^ GreenFast qPCR Blue Mix LR (AZ-2350, Azura Genomics Inc., Raynham, MA, USA). Amplification was carried out in a C1000^™^ Thermal Cycler (Bio-Rad Laboratories, Hercules, CA, USA) and the cycling conditions were the following: 3 minutes at 95°C for initial denaturation, 40 cycles of 10 sec at 95°C (denaturation), 30 sec at 60°C (annealing/extension) followed by melting curve analysis. The primers for phenotype markers and the housekeeping gene (Table 1) were purchased from Integrated DNA Technologies (Coralville, IA, USA). After normalization with 18S, relative gene expression was calculated using 2^−ΔΔCT^ method.

### 2.6 Statistical Analysis

Data were analyzed using GraphPad Prism 10 for Windows (version 10.3.0) and are represented as mean ± standard deviation. The normality of data was verified by Shapiro Wilk’s test. In MTT assays, viability of treated cells was compared to untreated cells using ordinary One-way Analysis of Variance (ANOVA) and Dunnet’s as posthoc test. In migration assays, the results were analyzed using ordinary Two-way ANOVA and Dunnet’s as posthoc test. Comparisons were performed to analyze the contribution to the variation of each EMF frequency. In qPCR analysis, fold change values obtained for each treatment were compared to untreated cells for each timepoint. The effect of EMF application was analyzed by comparing the results obtained in each frequency to no EMF-TNF-α treated cells. For all analysis a p-value < 0.05 was accepted as statistically significant. For several groups’ comparisons, differences were represented by *p <0.05, **p<0.01, ***p <0.001 and ****p <0.0001.

## 3. Results

### 3.1 Evaluation of viability in HMC3 cells:

**Figure 1:**
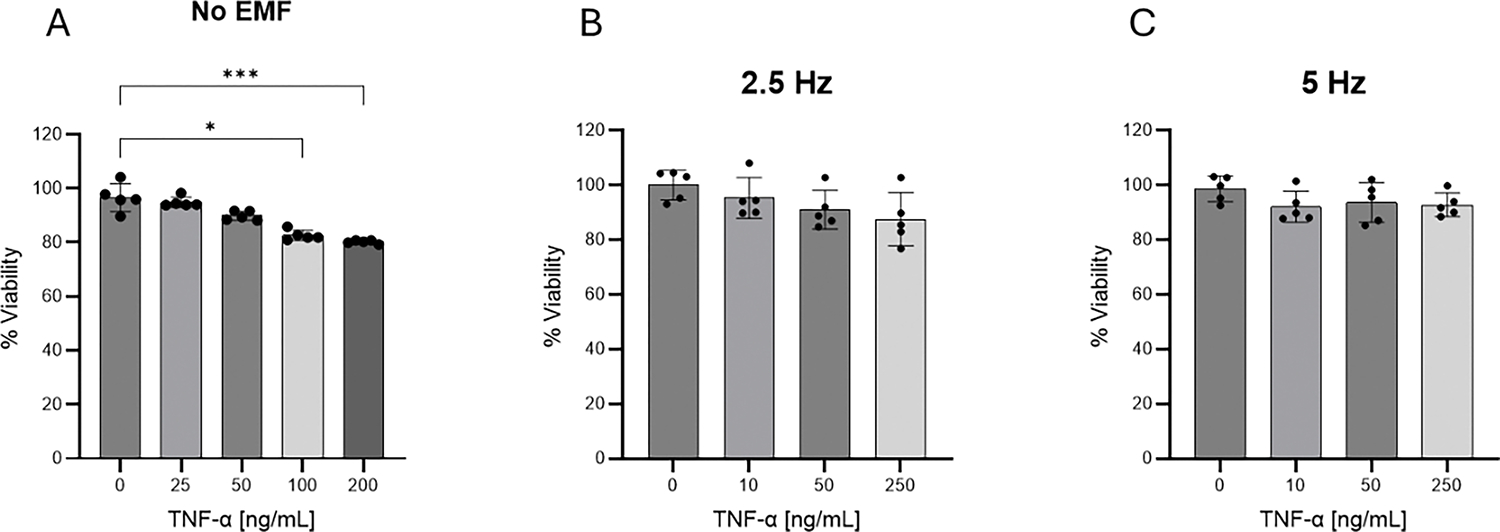
Evaluation of HMC3 cells viability by MTT assay. (A) Cells were exposed to increasing concentrations of TNF-α for 24 hours. To analyze the effect of EMF, cells were incubated with given concentrations of TNF-α during 20 min. After this time cells were exposed to EMFs of 2.5 Hz (B) or 5 Hz (C), 1 V intensity for 3 min and then returned to normal culture conditions of 37°C and 5% CO_2_. Values are representative from three different experiments and are presented as mean ± standard deviation. *p<0.05, ***p<0.001. TNF-α: tumor necrosis factor alpha; EMF: electromagnetic field.

Cellular viability was analyzed after 24 h exposure to increasing concentrations of TNF-α. As shown in Figure 1A, doses of 100 and 200 ng/mL reduced the viability of HMC3 cells. The application of 2.5 (Figure 1B) or 5 Hz EMF (Figure 1C) for 3 min did not affect cell viability. Taking these results into account, dose of 50 ng/mL of TNF-α was selected for further studies.

### 3.2 Effect of Electromagnetic field application on microglial migration:

**Figure 2:**
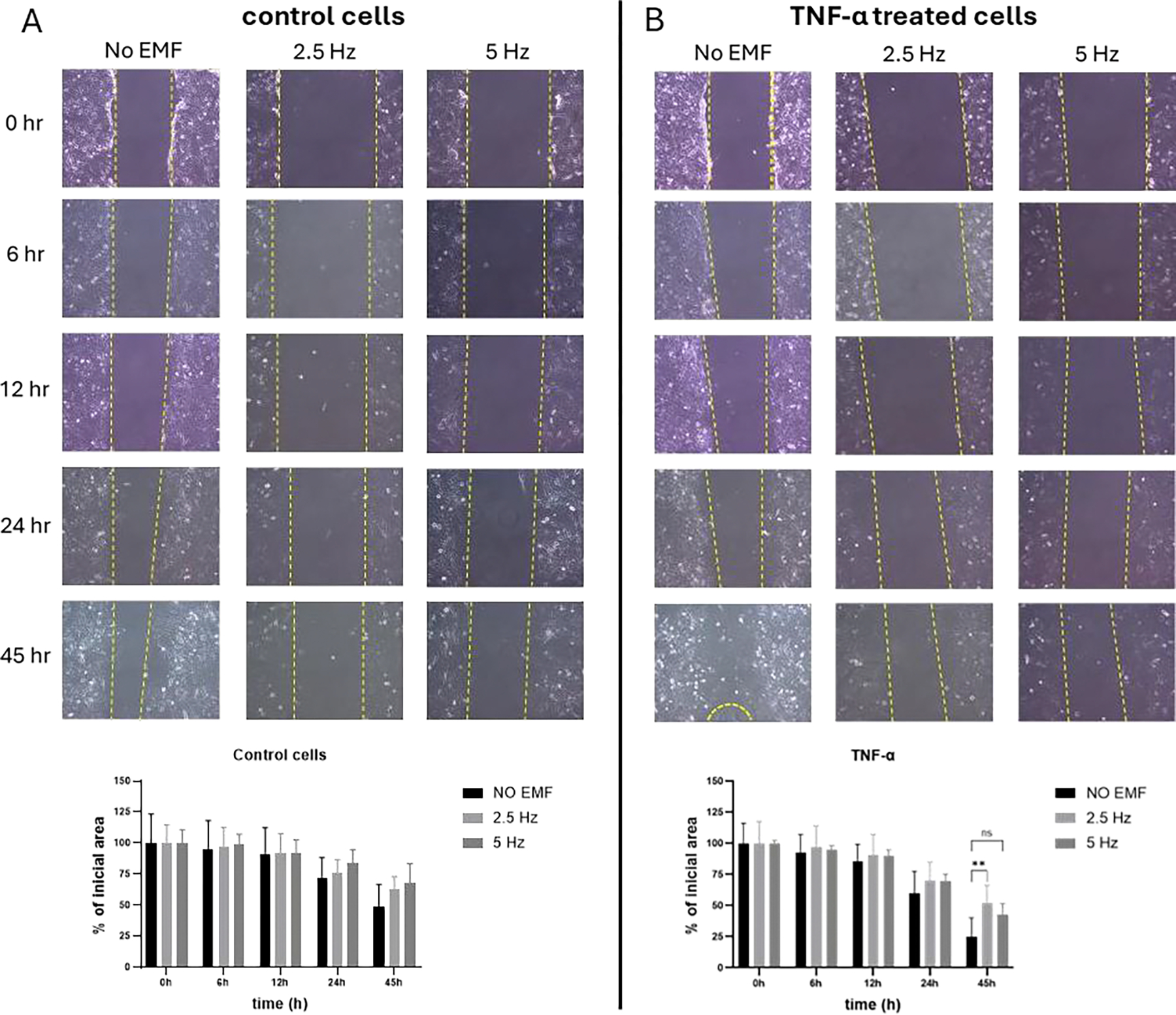
Effect of electromagnetic field (EMF) on cell migration in control (A) or TNF-α stimulated (B) HMC3 cells. Cells were treated with TNF-α (50 ng/mL) during 45 h. EMF with frequencies of 2.5 Hz or 5 Hz were applied for 3 minutes, 20 minutes after the addition of TNF-α. Migration was calculated as percentage of initial area, according to the formula described in Materials and Methods. The data are presented as the mean ± SD of three experiments. For each treatment and time point, comparisons were performed between each frequency and no EMF (**p<0.01). TNF-α: tumor necrosis factor alpha.

A scratch assay was performed to evaluate the effect of EMF application on the migration capacity of microglial cells in presence of pro-inflammatory stimulus. At 45 h, although not significant, a tendency to decrease or slow down migration was observed in non-stimulated cells exposed to EMF at both frequencies, compared to non-EMF control cells (Figure 2A). In cells treated with TNF-α, 2.5 Hz exposition significantly reduced the migration rate compared to non-EMF control cells (p=0.00058). Meanwhile, cells exposed to 5 Hz EMF showed a tendency to reduce their migration compared to non-EMF cells, with a p value=0.0597 (Figure 2B).

### 3.3 Effect of Electromagnetic field application on microglial phenotype:

**Figure 3:**
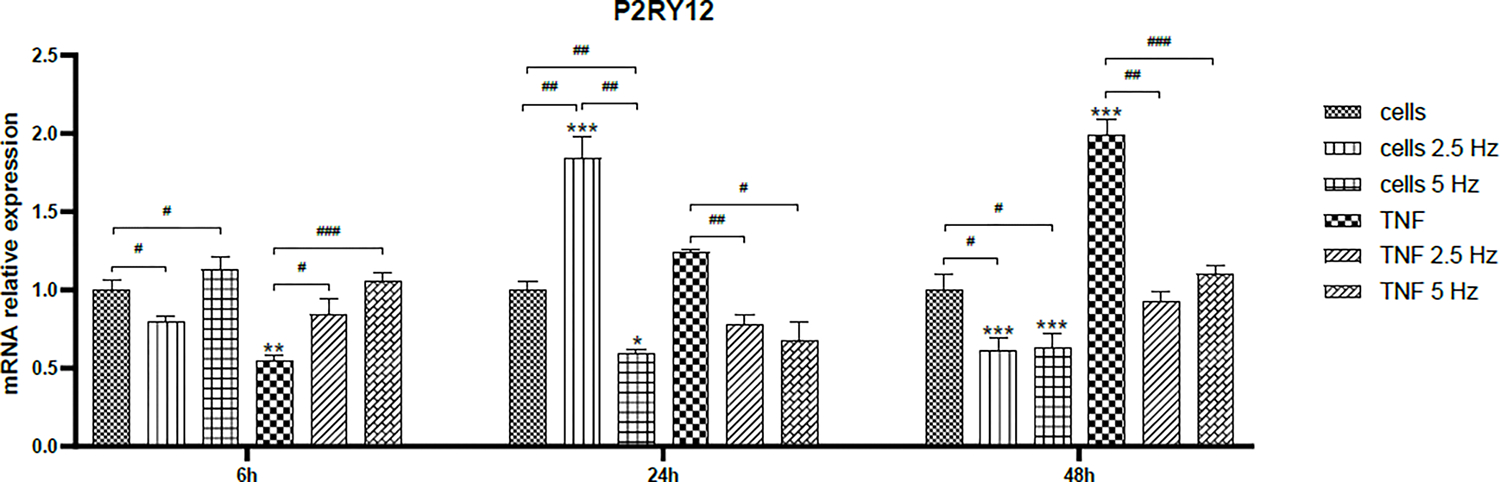
Effects of TNF-α and EMF exposition on the transcriptional expression of P2RY12 in HMC3 cells. Cells were incubated with TNF-α (50 ng/mL) and exposed to an (EMF) of 2.5 Hz or 5 Hz for 3 min. RNA samples were purified after 6, 24 and 48 h. TNF-α: tumor necrosis factor alpha; EMF: electromagnetic field; P2RY12: P2Y purinoceptor 12.

The effect of TNF-α treatment and EMF exposition on the transcriptional expression of microglial markers was assessed by RT-qPCR at 6, 24 and 48 h. Non-treated cells were used as controls to analyze the fold change. In all graphics, asterisks represent the difference between the given condition to control cells for each individual time point. Differences among non-EMF and EMF-treated cells are represented by number signs. Firstly, we analyzed the expression of the microglial marker P2RY12. At 6 h, TNF-α significantly reduced mRNA transripts of P2RY12, but the exposition to EMF restored the levels to normal (Figure 3). At 24 h, 2.5 Hz EMF significantly increased the expression in control cells and 5 Hz diminished it. In TNF-α treated cells, both frequencies significantly reduced the expression of P2RY12, and this effect was maintained until 48 h. In general, it was observed that TNF-α tended to increase the expression of P2RY12 in time, while EMF application restored the expression values to the normal physiological level on non-treated cells (Figure 3).

**Figure 4:**
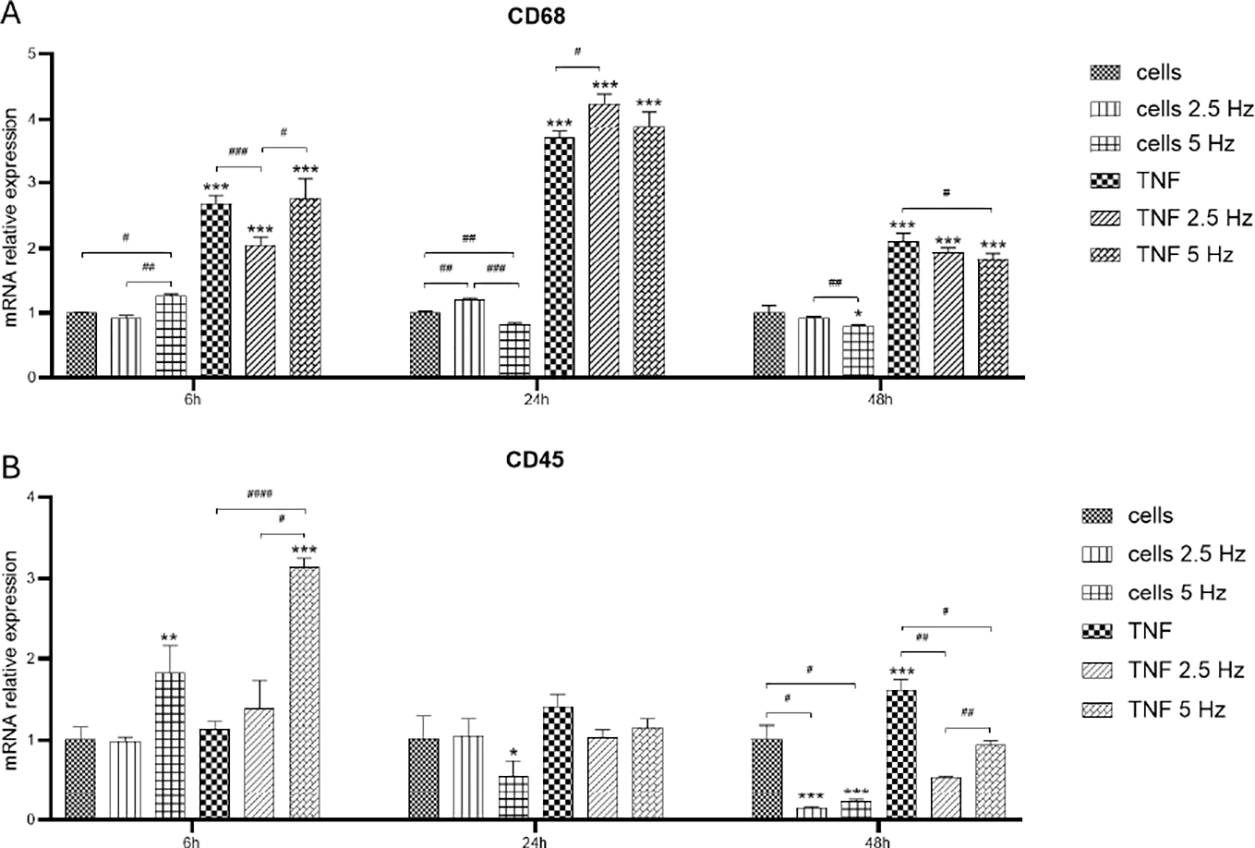
Effects of TNF-α and EMF exposition on the transcriptional expression of CD68 (A) and CD45 (B) in HMC3 cells. Cells were incubated with TNF-α (50 ng/mL) and exposed to an (EMF) of 2.5 Hz or 5 Hz for 3 min. RNA samples were purified after 6, 24 and 48 h. TNF-α: tumor necrosis factor alpha; EMF: electromagnetic field; CD68: cluster of differentiation 68 or microsialin; CD45: cluster of differentiation 45 or protein tyrosine phosphatase, receptor type, C (PTPRC).

Expression of non-exclusive microglial markers CD68 and CD45 was also analyzed. We observed a high induction of CD68 mRNA transcripts in all TNF-α treated cells as fast as 6 h, with the highest levels at 24 h (Figure 4A). At 6 h, the 2.5 Hz EMF exposition significantly reduced CD68 level compared to non-EMF, diminishing the pro-inflammatory effect of TNF-α. The other reducing influence of EMF on TNF-α effect was observed at 48 h in 5 Hz exposed cells (Figure 4A). Regarding CD45, at 6 h we observed an inducing effect of 5 Hz exposition on both control and TNF-α treated cells. After 24 h, the expression level of this marker was reduced to values similar to control cells. At 48 h, the reducing effect of EMF exposition at both frequencies was evident for control and TNF-α treated cells (Figure 4B).

**Figure 5:**
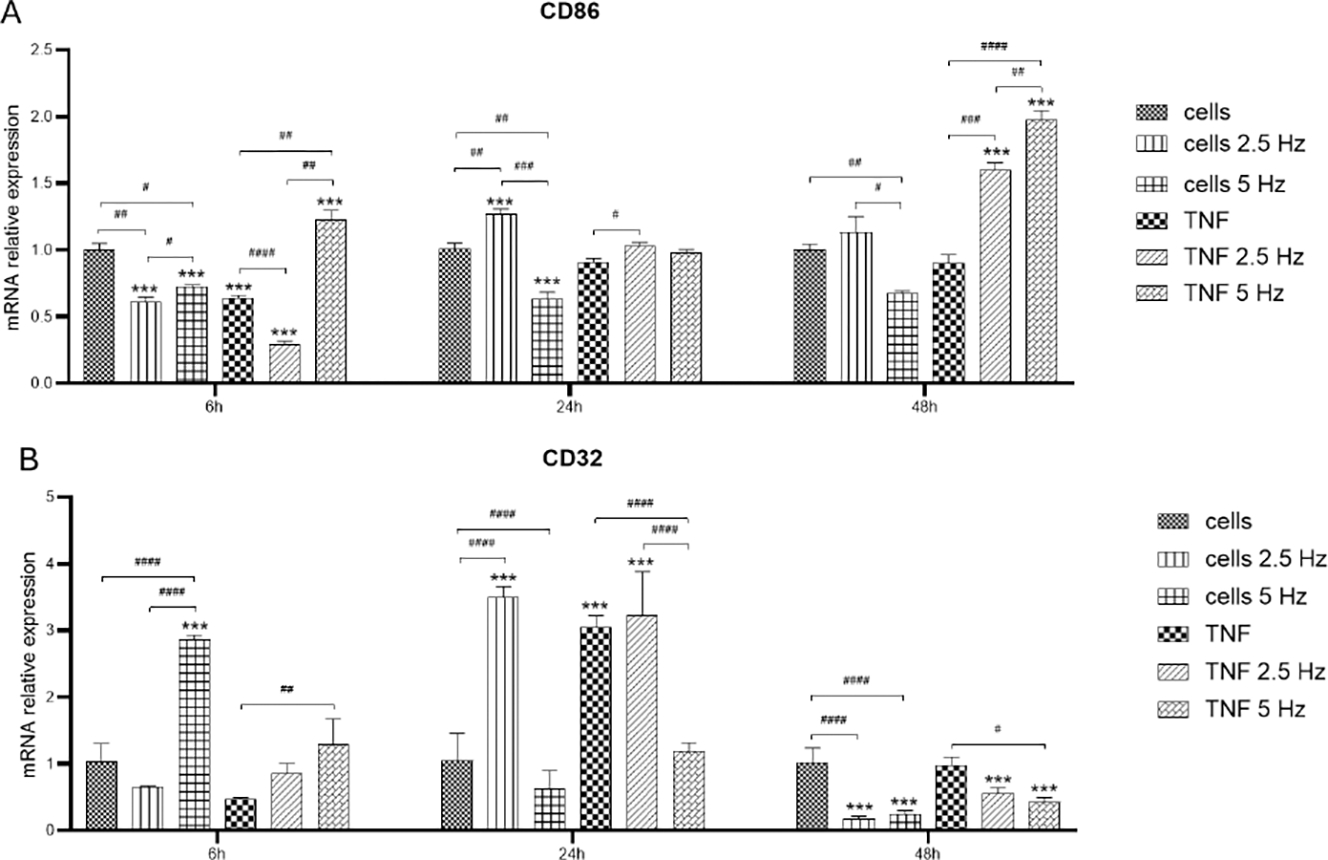
Effects of TNF-α and EMF exposition on the transcriptional expression of pro-inflammatory M1 phenotypic markers CD86 (A) and CD32 (B) in HMC3 cells. Cells were incubated with TNF-α (50 ng/mL) and exposed to an (EMF) of 2.5 Hz or 5 Hz for 3 min. RNA samples were purified after 6, 24 and 48 h. TNF-α: tumor necrosis factor alpha; EMF: electromagnetic field; CD86: cluster of differentiation 86 or T-lymphocyte activation antigen CD86; CD32: cluster of differentiation 32 or low-affinity immunoglobulin gamma Fc region receptor II phosphatase, receptor type, C (PTPRC).

To evaluate the transition to M1 phenotype we analyzed the expression of CD86 and CD32. Marker CD86 did not show high levels of induction after the treatment with the pro-inflammatory molecule TNF-α during the first 24 h. There was a significant increase in CD86 transcripts at 48 h in the cells incubated with TNF-α and exposed to both frequencies of EMF (Figure 5A). CD32 followed the same pattern of expression as P2RY12. At 6h, EMF at 5 Hz increased the level of this marker. Levels in TNF-α treated cells remained low. At 24 h, there was a significant increase in both types of cells exposed to 2.5 Hz and it was observed that there was strong stimulation in cells incubated with TNF-α. At 48 h, the level of expression of CD32 decayed in all experimental conditions, with a crystal-clear reduction effect of EMF in both control and TNF-α treated cells (Figure 5B).

**Figure 6:**
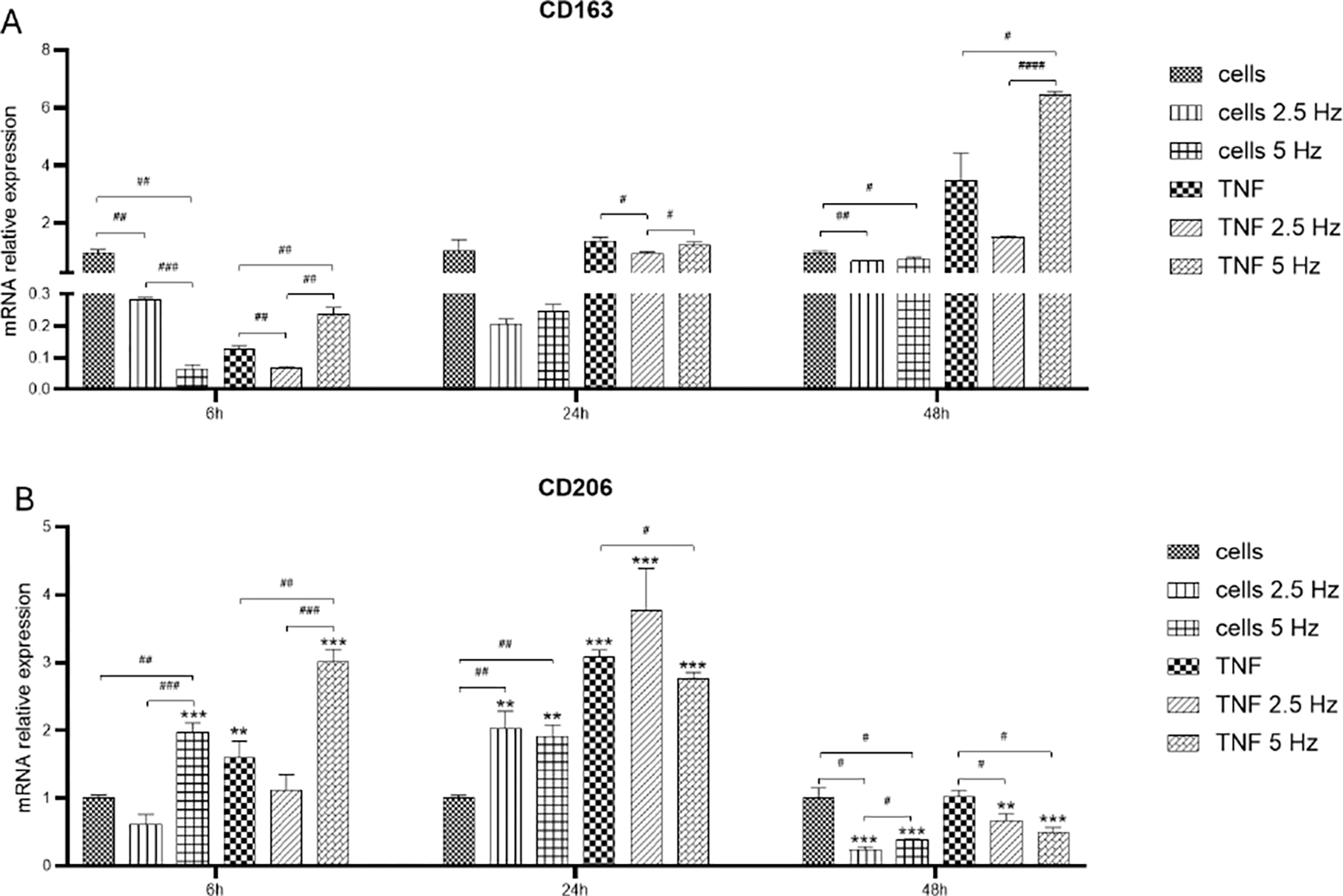
Effects of TNF-α and EMF exposition on the transcriptional expression of anti-inflammatory M2 phenotypic markers CD163 (A) and CD206 (B) in HMC3 cells. Cells were incubated with TNF-α (50 ng/mL) and exposed to an (EMF) of 2.5 Hz or 5 Hz for 3 min. RNA samples were purified after 6, 24 and 48 h. TNF-α: tumor necrosis factor alpha; EMF: electromagnetic field; CD163: cluster of differentiation 163 or scavenger receptor cysteine-rich type 1 protein; CD206: cluster of differentiation 206 or macrophage mannose receptor 1.

We also explored the effect of EMF on the expression of anti-inflammatory M2 markers CD163 (Figure 6A) and CD206 (Figure 6B). At 6 h, TNF-α reduced the levels of CD163, but the exposure to 5 Hz EMF significantly increased this marker. At 24 h, the expression of all experimental conditions was similar to the control cells. Instead, at 48 h, TNF-α treated cells showed higher expression of CD163 compared to control, having cells exposed to 5 Hz EMF the highest expression level (Figure 6A). After 6 h, the expression of CD206 was significantly induced after 5 Hz EMF application in both control and TNF-α treated cells (Figure 6B). At 24 h, all experimental conditions exhibited higher values compared to control cells, especially TNF-α treated cells. After 48 h, the expression levels in cells exposed to EMF were lower than that of control cells, irrespective of the frequency (Figure 6B).

## 4. Discussion

Inflammation is a key process during the initial stage of TBI. It is considered a protective response where the activation of immune cells and the release of pro-inflammatory molecules pave the way to remove cellular debris and to stimulate tissue repair. In this context, microglial cells are first class actors as they constitute the primary immune cells in the brain. According to the signals they receive, microglial cells can induce a beneficial response by releasing ani-inflammatory molecules to regain brain homeostasis, or they can enhance the neurotoxicity by secreting pro-inflammatory molecules to the extracellular milieu [29]. That is why it is important to find proper treatments or procedures that enhance the anti-inflammatory pro-repair microglial phenotype to attenuate the deleterious impact of TBI, and promote faster recovery of the patient. In that sense, our group recently published encouraging results regarding the application of EMF in an animal model of TBI in swine [19–21]. To go further in the characterization of EMF effect on specific cell types in the brain, we performed the present *in vitro* study, in which HMC3 microglial cells were incubated with TNF-α and exposed to EMF. Although with limitations, our results contribute to shedding light on what could be occurring *in vivo* after the application of EMF.

TNF-α is one of the main cytokines released by the brain after a mechanical trauma [24]. It has been shown that excessive activation of TNF-α triggering pathways results in cell death, damage to the blood–brain barrier, neuronal loss, cognitive decline, and long-term neurological complications [30]. On the other hand, TNF-α has been used in numerous studies *in vitro* as a pro-inflammatory stimulus for microglia [31]. Taking these elements into account, we decided to use TNF-α to recreate the pro-inflammatory scenario in our study. The TNF-α dose of 50 ng/mL was employed as it was demonstrated that it does not affect cellular viability. According to our results, EMF exposure helped reverse the loss of cell viability observed when incubating cells with the highest doses of TNF-α (Figure 1). In the context of TBI, preserving microglia viability would contribute to ensuring an adequate immune response and favor the shift toward a reparative phenotype.

Another key characteristic of microglia is its ability to migrate towards the injured areas. After damage, secreted chemokines recruit microglia towards the site of injury, and they respond by secreting molecules to stimulate the recruitment of other immune cells to the compromised area [32]. That is why it has been proposed that increased migration may also be a feature of activated microglial cells [33]. It is well stablished that TNF-α activates signaling pathways involved in microglial migration, and also induces changes in the cytoskeleton of microglial cells, facilitating their movement [34]. As observed in our study, TNF-α significantly induced migration of microglial cells, but the exposure to EMF, particularly 2.5 Hz, significantly reduced this ability. This effect has been observed related to some pharmacological interventions like treatment with efonidipine, a calcium channel blocker [35] or with JQ1, an epigenetic agent that lowers inflammation [36]. The reduction of microglial cell migration after EMF exposure might occur as a direct effect of EMF on the genes involved in cell migration or indirectly as a consequence of the inhibition of pro-inflammatory pathways. According to our previous results, EMF stimulation is associated with a reduction of inflammatory markers at the transcriptional and translational levels *in vivo* [19]. Further studies must be accomplished to elucidate both theories.

Microglia is considered a very heterogeneous group of cells in terms of density in the different brain areas, morphology and protein markers expression. Although each microglial phenotype has a specific proteomic profile [37], transcriptomic analysis revealed that activated microglia rarely fit the classification perfectly, as usually a mixed microglial population is observed. Sometimes, quantitative expression of a given marker, not only presence/absence, helps to recognize resident microglia from peripheral immune cells and to differentiate one phenotype from the other, which may be indicative of different functional states. In the present work, we aimed to evaluate the effect of the exposure to EMF on the expression of M1/M2 phenotypic markers. We started analyzing the purinergic receptor P2RY12, considered one of the most specific general microglial markers [7]. This belongs to the family of purinergic receptors that are activated by extracellular adenosine triphosphate (ATP) released from damaged cells, acting as a danger signal that triggers microglial activation and neuroinflammation [38]. It has been informed that P2RY12 plays a major role in microglial chemotaxis in response to local CNS injury [39]. In our study, it is clearly observed that TNF-α induces the expression of this marker, as increasing levels are shown from 6 to 48 h. The application of EMF significantly reduced the transcriptional expression of this receptor 24 and 48 h after the treatment (Figure 3). This effect could be beneficial as it has been shown that blocking the activation of P2Y receptors reduces neuroinflammation and blood-brain barrier disruption in animal models of TBI [40].

CD68 was another marker we analyzed. This molecule, also known as macrosialin, is a transmembrane protein localized in cellular, lysosomal, and endosomal membranes of monocytes and macrophages/microglia. It is strongly upregulated during inflammation [41], as it was observed in our study, where TNF-α highly induced its expression, as earlier as 6 h after the addition of treatment. The highest expression levels were observed at 24 h, while there was a reduction at 48 h, but still higher compared to control cells. The application of the EMF did not exert a prominent effect on this marker, although a slight decrease was observed at 48 h when applying the 5 Hz EMF.

CD45, also known as receptor-type tyrosine-protein phosphatase C (PTPRC) is a positive regulator of T-cell activation [42] and another non-exclusive marker for microglial cells. It has been informed that CD45 expression increases in microglia upon inflammation [43]. In our study, the expression of CD45 also increased in time in response to TNF-α (Figure 4B). The application of EMF, especially at 2.5 Hz, considerably reduced the transcriptional levels of this marker. This lowering effect might be considered helpful as it could be associated with a less activated state of microglia, which can be beneficial in reducing chronic inflammation and neurodegeneration.

Regarding M1 phenotypic markers, we analyzed CD68, also known as T-lymphocyte activation antigen CD86, as it is activated in M1 microglia. In our study, TNF-α did not stimulate the expression of CD86 above the levels observed in control non-treated cells. However, the application of EMF in presence of TNF-α highly increased the expression of this marker 48 h after the exposure (Figure 5A). We also evaluated the expression of CD32, a membrane receptor for the Fc region of IgG. It has been shown that the expression of this marker increases in response to immune complexes and pro-inflammatory cytokines like IFN-γ [10]. In our study, a significant induction of CD32 was observed 24 h after treatment with TNF-α. After that, the expression level went back to normal. Regarding EMF effect, we observed that, at 6h, 5 Hz EMF exerted a strong induction of CD32 in both control and TNF-α treated cells. For 2.5 Hz EMF, this effect was delayed as it occurred at 24 h. Conversely, at 48 h, the application of EMF was associated with a significant reduction of CD32 expression (Fig. 5B), an effect that was also observed for P2RY12, CD45 and, in less extent, CD68.

Classical M2 phenotypic markers are CD163 and CD206 [44]. CD163 is a hemoglobin scavenger receptor responsible for clearing oxidative hemoglobin [12]. In our study, at 6 h there were very low expression levels of CD163 in cells incubated in TNF-α. No differences were observed after 24 h; but at 48 h, the exposure to 5 Hz EMF significantly increased the expression of this marker, suggesting a shift towards an anti-inflammatory phenotype. Meanwhile, the exposure of cells to the 5 Hz EMF induced the expression of CD206, an M2 phenotype marker also known as mannose receptor MRC1, which is involved in important cellular functions, especially in pinocytosis and phagocytosis [45]. This effect was observed in control and TNF-α treated cells. At 24 h, all experimental conditions showed higher levels compared to un-treated cells. However, after 48 h, levels of this marker went back to normal and the exposure to EMF significantly reduced its expression.

Microglia comprise a highly dynamic group of cells, in which markers of the classical M1 and M2 groups can coexist, suggesting the presence of numerous intermediate phenotypes. The predominance of one phenotype over another depends on all the signals present in the cellular environment, which vary according to the age of the individual or the presence/absence of neurological pathologies and/or damage. For example, in TBI there is a microglial phenotype known as Mtran in which the co-expression of TGF-β (M2 marker) and CD16 (M1 marker) is found [46]. Considering our results, we can summarize that at 6h, microglial cells exposed to TNF-α are mainly CD68^+^. The exposure to 5 Hz EMF increases the expression of CD45 and CD206. The co-expression of both markers is found in certain contexts, such as gliomas or retinal degeneration. These cells often play a role in phagocytosis and managing the immune response [47]. It has been hypothesized that the co-expression of these markers indicates a complex state of activation where microglia is involved in both inflammatory and reparative functions. Meanwhile, after 24 h of stimulation/exposure, CD68 is still high, and 2.5 Hz EMF stimulated the expression of CD32 and CD206. This combination is also suggestive of both immune response and tissue repair. For example, in models of retinal degeneration, microglial cells expressing both markers have been observed, suggesting their role in phagocytosis and managing inflammation [14]. Finally, at 48 h, levels of CD68 diminished and 5 Hz EMF induced the expression of CD86 and CD163. This dual expression is observed in various pathological conditions, such as during the early stages of hypertension or in response to certain inflammatory stimuli [48]. Specifically, CD163 is a marker for acquired deactivation of microglia and phagocytosis [49].

The evolution of the expression pattern of different markers in this study suggests that exposure to EMF, and more prominently to the frequency of 5 Hz, could be stimulating the phagocytic capacity of microglial cells. This activity, extrapolated to the *in vivo* scenario of TBI, could be highly beneficial during the first stages after the mechanical trauma in order to clear away dead cells, debris, and pathogens, preventing the accumulation of potentially toxic substances.

## 5. Conclusion

Microglial cells are highly dynamic and responsive to the inflammatory environment. Particularly, in the context of TBI, the activation of microglia is essential to perform key functions like clearing debris, fighting infections, and releasing pro-inflammatory mediators. Once the situation is controlled, the secretion of anti-inflammatory factors by microglia is mandatory to downregulate the immune response and promote the transition to the healing phase. Sometimes, the resolution of inflammation is not properly achieved, leading to chronic damage of CNS and the development of neuronal complications. In that sense, the use of pharmacological and non-pharmacological interventions has been pivotal. The application of low frequence EMF has shown important results in terms of reduction of pro-inflammatory markers in animal models of TBI. In the present study we analyzed the effect of EMF specifically on microglial cells. Although with the limitations of an *in vitro* approach, our results suggest that EMF stimulates the phagocytic capacity of microglial cells, considering the activation pattern of different microglial markers. The reduction in microglial migration exerted by EMF, even in the presence of TNF-α, could be a consequence of resolving the pro-inflammatory stimulus, paving the way to the transition towards a less migratory, homeostatic state. This shift is often accompanied by the adoption of anti-inflammatory or restorative phenotypes (CD206+ or CD163+), observe also as a result of EMF exposure. Further studies should be addressed to complete the characterization of EMF effect on microglial cells expanding the analysis to other molecules involved in the inflammatory response.

## Figures and Tables

**Table 1:** Sequences of forward and reverse oligonucleotides used for phenotypic characterization by RT-qPCR. 18S gene was used as a housekeeping gene to normalize results.

Phenotype marker	Forward	Reverse
P2RY12	5’-GTGATGCCAAACTGGGAACAGG-3’	5’-CTGGTGGTCTTCTGGTAGCGAT-3’
CD68	5’-CTCCAAGCCCAGATTCAGATT-3’	5’-GGGAATGAGAGAAGCAGGTG-3’
CD45	5’-GGTTTCAAAGAACCCAGGAAATAC-3’	5’-ACATCGAGTGACCATGACAATAA-3’
CD86	5’-CCCAGAACCTAAGAAGATGAGTG-3’	5’-GCTCGTAACATCAGGGAATGA-3’
CD32	5’-CTCTGGTCAAGGTCACATTCTT-3’	5’-TTGGATGAGAACAGCGTGTAG-3’
CD206	5’-GGACGTGGCTGTGGATAAAT-3’	5’-ACCCAGAAGACGCATGTAAAG-3’
CD163	5’-GTGTGATGACTCTTGGGACTT-3’	5’-CCTGACCAAACTCTGCTTCT-3’
18S	5’-CCCACGGAATCGAGAAAGAG-3’	5’-TTGACGGAAGGGCACCA-3’

CD: cluster of differentiation. General microglia markers: P2Y12: P2Y purinoceptor 12; CD68 or microsialin; CD45 or protein tyrosine phosphatase, receptor type, C (PTPRC). M1 phenotype: CD86 or T-lymphocyte activation antigen CD86; CD32 or low-affinity immunoglobulin gamma Fc region receptor II. M2 phenotype: CD206 or macrophage mannose receptor 1; CD163 or scavenger receptor cysteine-rich type 1 protein.
